# Interfacing Machine Learning and Microbial Omics: A Promising Means to Address Environmental Challenges

**DOI:** 10.3389/fmicb.2022.851450

**Published:** 2022-04-25

**Authors:** James M. W. R. McElhinney, Mary Krystelle Catacutan, Aurelie Mawart, Ayesha Hasan, Jorge Dias

**Affiliations:** ^1^Applied Genomics Laboratory, Center for Membranes and Advanced Water Technology, Khalifa University, Abu Dhabi, United Arab Emirates; ^2^Department of Biomedical Engineering, Khalifa University, Abu Dhabi, United Arab Emirates; ^3^EECS, Center for Autonomous Robotic Systems, Khalifa University, Abu Dhabi, United Arab Emirates

**Keywords:** machine learning, microbial ecology, metagenomics, environmental monitoring, microbiology, artificial intelligence, microbial omics, predictive modeling

## Abstract

Microbial communities are ubiquitous and carry an exceptionally broad metabolic capability. Upon environmental perturbation, microbes are also amongst the first natural responsive elements with perturbation-specific cues and markers. These communities are thereby uniquely positioned to inform on the status of environmental conditions. The advent of microbial omics has led to an unprecedented volume of complex microbiological data sets. Importantly, these data sets are rich in biological information with potential for predictive environmental classification and forecasting. However, the patterns in this information are often hidden amongst the inherent complexity of the data. There has been a continued rise in the development and adoption of machine learning (ML) and deep learning architectures for solving research challenges of this sort. Indeed, the interface between molecular microbial ecology and artificial intelligence (AI) appears to show considerable potential for significantly advancing environmental monitoring and management practices through their application. Here, we provide a primer for ML, highlight the notion of retaining biological sample information for supervised ML, discuss workflow considerations, and review the state of the art of the exciting, yet nascent, interdisciplinary field of ML-driven microbial ecology. Current limitations in this sphere of research are also addressed to frame a forward-looking perspective toward the realization of what we anticipate will become a pivotal toolkit for addressing environmental monitoring and management challenges in the years ahead.

## Introduction

Expansion of the human population is increasing resource consumption and discharge of waste products, placing significant burdens on the biosphere (Burrell et al., 2020; Grantham et al., 2020; Lv et al., 2020; Albert et al., 2021; Lu et al., 2021; Naumann et al., 2021; Ortiz-Bobea et al., 2021). These activities are contributing to the multifaceted pollution of the global ecological systems (Julinová et al., 2018; Santos et al., 2019; Turan et al., 2019; Vardhan et al., 2019; Briffa et al., 2020; Pulster et al., 2020; Simul Bhuyan et al., 2021; Sohrabi et al., 2021; Li and Fantke, 2022). Consequently, we are witnessing an accelerating loss of biodiversity, habitats, and climate change (Sintayehu, 2018; Brühl and Zaller, 2019). Gauging and forecasting such anthropogenic environmental impacts is often limited in scope due to scale-up challenges. At large scale, this endeavor remains an inordinately complex and resource-intensive task and therefore represents a major scientific goal.

At 93 gigatons carbon (Gt C), microbial communities comprise approximately 20% of the total estimated global biomass and exclusively form the deep subsurface biome (estimated at 70 Gt C) (Bar-On et al., 2018). These communities are ubiquitously distributed across the biosphere where their activities are central in shaping the environments of our planet (Gibbons and Gilbert, 2015); microbial communities possess exceptionally broad metabolic capabilities, enabling their utilization of many xenobiotics (Katsuyama et al., 2009; Junghare et al., 2019). Microbes can have short generation times and are amongst the first responders with perturbation-specific cues and markers (De Anda et al., 2018; Astudillo-García et al., 2019) these can therefore serve as a valuable source of biological information for establishing the status of their respective environmental niches and can serve as dynamic biosensors for monitoring and tracing environmental changes (Cesare et al., 2020; Morimura et al., 2020).

**FIGURE 1:**
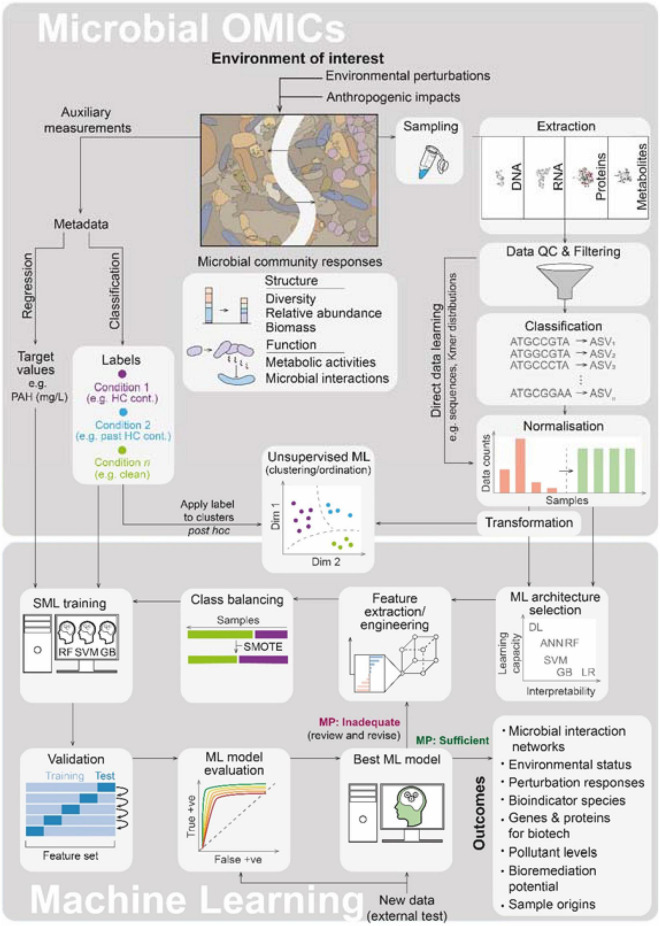
The interface of microbial omics and machine learning (ML). A generalized and simplified overview of the workflows is presented highlighting the major steps in the microbial omics and ML workflows as they relate to one another along with key outcomes obtainable from the application of ML to omics data. Microbial community responses (biological information on which learning is aimed) are summarized below the cartoon snapshot of a contaminated environment of interest. Here, HC cont., hydrocarbon contamination; PAH, polyaromatic hydrocarbons (as examples of targets in petroleum hydrocarbon scenarios); QC, quality control; ASV, amplicon sequence variant (ASVs are given here as an example of an omics classification, other examples include the often used OTU, genes, mRNA transcripts, protein categories or metabolite IDs); DL, deep learning; ANN, artificial neural networks (shallow); RF, random forest; SVM, support vector machine; GB, gradient boost; LR, logistic regression; SMOTE, synthetic minority oversampling technique; SML, supervised machine learning; and MP, model performance.

Omics methodologies enable rapid community-wide profiling of microbial populations across environmental perturbations. Omics data are information-rich, leading to an unprecedented volume of large multidimensional data sets with potential for predictive environmental classification and forecasting. However, the inherent complexity in these data conceals the patterns underlying the biological information, challenging manual curation and interpretation. Machine learning (ML) is well suited to address such challenges and there has been a sharp rise in their application in health-oriented microbiomics (Zeller et al., 2014; Szafrański et al., 2015; Knight et al., 2018). ML-driven omics is now being applied to address environmental challenges (Figure 1). Here, we will discuss the state of the art in this interdisciplinary field and highlight considerations, ongoing limitations, and challenges for future work. The interface between ML and molecular microbial ecology (MME) holds great promise for significantly advancing environmental monitoring and management practices. Indeed, ML will likely become a routine toolkit for the molecular microbiologist and will be essential to manage large multidimensional environmental omics data.

## Main Body

### A Primer on Machine Learning

Machine learning approaches can be supervised (SML) or unsupervised (USML). In SML methods, data sets are reduced/converted into the sets of features which serve as the input and form a variable for the SML model. Features are measurable and informative properties of the data, e.g., taxa abundances, annotated with metadata of interest (labels) which define the desired output (the target). Feature sets are subset into groups for model training and model testing/validation for SML learning. The SML architecture then attempts to derive a model that can predict the label for new input data. SML can be carried out to address regression or classification challenges. For regression, the SML tool predicts values for a continuous series (such as levels of environmental pollutants). For classification, the SML will predict the conditional label pertaining to the sample (such as contamination status). Deep learning (DL) is a subset of SML, which employs neural networks with multiple (>3) processing layers and has the highest capacity for learning. For USML, no label or target output is defined; instead the USML architecture establishes patterns in the data naively, usually by clustering or ordination projections. USML is particularly useful for exploratory analysis of microbial omics data and includes ordination methods that are commonly applied in microbiology. Here we focus primarily on SML applications for environmentally centered microbial omics research. For more details on the underlying principles of ML for microbial ecology, readers are encouraged to see reviews (Ghannam and Techtmann, 2021; Goodswen et al., 2021).

### Omics Data Sets Are Rich in Learnable Biological Information

Anthropogenic perturbations give rise to spatiotemporal patterns in microbial communities by influencing the following: abundances, interactions between, and dispersal of community members (Blaser et al., 2016; Liao et al., 2018). Community dynamics are perturbation-specific, reproducible, and predictable, affecting taxonomic diversity, differential abundances in taxa, functional gene clusters, and shifts in metabolic circuits which influence microbial interactions (Figure 1). Microbial omics approaches are rapidly advancing our views of these complex shifts and have opened myriad avenues for the utilization of microbial data to address environmental challenges. Often these omics approaches scrutinize a single systems level (e.g., DNA or RNA), but can synergistically provide more information when integrated with supporting omics data from other systems layers (Franzosa et al., 2015). Such integrative omics represents a powerful means to understand communities through cross-systems-level descriptions but is in its infancy and yet to be much applied in this area. A central challenge for any ML-led omics analyses is the preservation of the biological information hidden within the microbial community, throughout the workflow (Figure 1), to allow for effective learning. There are numerous ways *via* which the biological information in omics samples can be compromised. These pitfalls occur at virtually all decision points in the omics workflow and begin with the experimental design phase. The significance of a given pitfall is highly dependent on the phenomena under investigation and aims of the study but common pitfalls include inadequate sampling, improper preservation, sample transport conditions or subcommunity sampling (e.g., planktonic/sessile), biases arising from sample handling (e.g., during extraction and amplification), the choice of sequencing/liquid chromatography-mass spectrometry (LC–MS) platform and analytical methodology, classification and filtering of omics data (which can remove rare but important taxa, transcripts, or proteins), artifacts from data transformation and normalization approaches (correcting for library size is especially essential for meta-analyses), and the choice and engineering of features. A number of considerations can help in preserving the biological information for omics-led SML, and many are discussed in the following.

### Workflow Considerations

#### Microbial Omics Input

Microbial omics pitfalls, from sampling to the bioinformatics pipeline, can reduce or bias the information yielded (Gutleben et al., 2018; Kaster and Sobol, 2020). Typically, some trade-off must be made in the experimental design, for which options have been suggested (Franzosa et al., 2015). In metataxonomics, resolution is usually limited to the genus level, though it is the most commonly used omics input for SML (Table 1), wherein relative operational taxonomic unit (OTU) abundances form the feature set (Miao et al., 2020; Janßen et al., 2021; Kim and Oh, 2021). However, the use of OTUs is inherently limiting for retaining community information and can miss important taxonomic groups. Indeed, since the development of the more biologically meaningful amplicon sequence variants (ASVs; Callahan et al., 2017), the absence of ASVs in most metataxonomic studies is striking. As ASVs represent a more accurate basis for taxa assignment, it will be interesting to see how their application influences ML performances in future.

**TABLE 1:** Example applications of the SML of microbial Omics data for addressing environmental challenges.

Environment	Niche	Application	Omics	Input data	Feature	Target(s)	SML architectures	Software	References
Aquatic	Marine (Coral Reef)	Prediction of environmental status	metataxonomics	16S rRNA OTUs	OTU abundance	Eutrophication indicators and temperature	RF	Caret and RF R packages	Glasl et al., 2019
Industrial	WWTP	Prediction of environmental variable to identify key subpopulations	metataxonomics	16S rRNA OTUs	OTU abundance, PCA coordinates	WWTP water temperature	LR, RF, SVML, DT, KNN, SVMRBF	Scikit-Learn	Kim and Oh, 2021
Terrestrial	Soil^1^	Prediction of carbon cycling	metataxonomics	16S rRNA OTUs	OTU abundance	[DOC]	RF, ANN	THEANO, Scikit-Learn	Thompson et al., 2019
Terrestrial	Compost	Classification of microbial biomarkers	metataxonomics	16S rRNA OTUs	OTU abundance	Compost cycle	RF	RF R package	Zhang et al., 2020
Terrestrial	Ground water + Soil^1^	Prediction of environmental contaminants	metataxonomics	16S rRNA OTUs	OTU abundance	[dioxane] and [CVOCs]	RF		Miao et al., 2020
Terrestrial	Soil	Prediction of environmental quality	metataxonomics	16S rRNA OTUs	OTU abundance	Soil physicochemical features	RF	RF R package	Hermans et al., 2020
Aquatic	Marine (coastal waters)^1^	Prediction of environmental contaminants	metataxonomics	16S rRNA OTUs	OTU abundance, 16S rRNA gene sequences	Glyphosate	RF, ANN	RF R package and DL4J	Janßen et al., 2019
Aquatic	Freshwater (river)	Classification of anthropogenic pathogen loads	metataxonomics^2^	16S rRNA OTUs	OTU abundance	Fecal source	RF, MCMC	RF R package and SourceTracker	Dubinsky et al., 2016
Aquatic	Marine and Freshwater	Classification of microbial biomarkers	metataxonomics	16S rRNA and ITS OTUs	OTU abundance	Plastisphere communities	RF	RF R package	Li et al., 2021
Aquatic	Marine sediment (munitions dumpsite)	Prediction of environmental contaminants	metataxonomics	16S rRNA OTUs	OTU abundance	TNT	RF, ANN	Ranger R package ANN R keras framework + TensorFlow back end	Janßen et al., 2021
Aquatic	Freshwater (river)	Classification of sample origin	metataxonomics	16S rRNA OTUs	OTU abundance (top taxa)	Sample origin	RF	RF R package	Wang et al., 2021
Aquatic	Marine (oceanic waters)	Classification of trophic modes	Metatranscriptomics	Gene expression levels	expression levels of selected Pfam entries	Trophic mode (photo/hetero/mixo)	RF, DT, ANN	NR and XGBoost	Lambert et al., 2021
Terrestrial	Soil	Prediction of crop productivity	metagenomics	Shotgun sequencing	OTU abundance	Crop productivity	RF	Ranger R package	Chang et al., 2017
Terrestrial	Soil	Prediction of soil phylogroups from environmental metadata	metagenomics	NR	NR	*Listeria* species	RF	RF R package	Liao et al., 2021

*^1^Indirectly studied in microcosms.*

*^2^Using PhyloChip array.*

*Here, ANN, Artificial Neural Network; CVOCs, Chlorinated Volatile Organic Compounds; DOC, Dissolved Organic Carbon; DT, Decision Tree; KNN, K-Nearest Neighbors; LR, Logistic Regression; MCMC, Markov Chain Monte Carlo; NR, Not reported; RF, Random Forest; SVML, Support Vector Machine (SVM) with a linear kernel; SVMRBF, SVM with a radial basis function kernel; TNT, trinitrotoluene; WWTP, Wastewater Treatment Plant.*

Metagenomics is highly sensitive for low-abundance taxa, but is rarely applied for SML and carries additional costs which may limit sampling and options for ML (Chen and Tyler, 2020). Importantly, metagenomic approaches do not always convey a clear advantage over the more cost-effective metataxonomic approach (Xu et al., 2014). The choice between metataxonomics and metagenomics is evidently not clear-cut and should be considered in light of the expected community under study, choice of sequencing platform, and research goals. Microbial omics inputs are most often derived from closed-reference databases, leading to inevitable loss of learnable biological information in environmental samples due to unclassified/misclassified data (Chen and Tyler, 2020). However, the development of ML and DL tools (Liang et al., 2020) for enhancing taxonomic classification in metagenomic data sets could prove helpful. Alternatively, the direct use of biological sequences (from microbial omics surveys) circumvents this issue (by forgoing categorical assignment), thereby permitting the inclusion of more comprehensive feature spaces, at the cost of reducing the immediate interpretability for the user. Informative abstractions of omics data, such as the use of K-mer distributions as a feature set, have shown success in both taxonomic (Fiannaca et al., 2018) subtyping (Solis-Reyes et al., 2018) and phenotypic (Aun et al., 2018) classification, and are applicable to environmental applications. Indeed, K-mer abstractions have shown predictive potential for classifying sample environment and host-phenotype (an environmental status) that excels over OTU features (Asgari et al., 2018). Environmental metatranscriptomics-led SML is currently limited. However, the approach has been shown to uncover the mixotrophic processes of protists in response to nutrient gradients in the Pacific Ocean (Lambert et al., 2021), thereby demonstrating that trophic modes can be readily predicted from metatranscriptomic data.

#### Choice of Machine Learning Architecture

There is a broad selection of the SML tools to select from and each carries its own advantages and limitations (Goodswen et al., 2021). Not a single architecture performs best in all environmental application cases and users must make a trade-off in terms of interpretability, learning performance, computational costs, data requirements, and ease of implementation (Ghannam and Techtmann, 2021). At the outset, selecting a set of architectures can help to ensure the delivery of research goals. Random forest (RF) is a popular choice for microbial omics-driven SML for its learning capacity, straightforward implementation, and high degree of interpretability (Ghannam and Techtmann, 2021). For especially complex tasks, or where knowledge is limited, DL approaches (multi-layered architectures) have the highest performance, as they can self-learn (i.e., do not require user extraction of) the feature set (Christin et al., 2019). However, DL comes with elevated computational costs and low interpretability of the underlying model (“*black box*” effect) and requires large volumes of data (thousands of samples). Consequently, though very promising, DL approaches for environmental omics are currently limited.

#### Feature Engineering

Feature selection and engineering are crucial for generating meaningful SML-based ecological models. Reducing the feature space can help to limit overfitting, reduce computational costs, improve cross-study comparison, and improve generalized prediction performance across data sets (Ghannam and Techtmann, 2021). However, care is needed when reducing features for training as biologically meaningful features can be missed if feature selection is based on abundance. This is especially so when assessing anthropogenic perturbations of pollutants in the environment, wherein the rare microbiome (taxa representing <0.1% of the total community) comprise a significant reservoir of gene clusters that enable the utilization and degradation of xenobiotic organic compounds (Wang et al., 2017). Taking embedded approaches for feature selection (that can evaluate across the full feature space) (Wang et al., 2017) or a biologically driven feature selection method (such as taxonomically aware hierarchical feature engineering) (Oudah and Henschel, 2018) may help in optimizing feature selection in metataxonomics-driven ML applications. Feature selection methods designed for functional feature sets are still notably lacking in this space.

Conventional statistics require assumptions on the underlying data and care is needed, given the compositional nature of microbial omics data sets (Gloor et al., 2017). For example, conventional ecological models often assume monotonicity in relationships, which can hinder ecological explanations of community variance across study sites. By applying SML (allowing for non-monotonic feature capture), the ability to capture this variance can increase nine-fold (Fontaine et al., 2021). It is important to note that the goal of SML should not be to replace classical statistical modeling, but rather to complement it. Integrating these two approaches presents an promising opportunity to leverage their advantages for predictive environmental microbiology (Lopatkin and Collins, 2020) and monitoring. For multi-omics studies, feature selection and engineering becomes increasingly complex with the successive systems levels, and there is much to be done in this area. In such studies, functional data across systems levels will likely need to be empirically assessed prior to SML to identify the most informative biomarkers for learning (Xu et al., 2014).

#### Evaluating Data Leakage

Data leakage is a subtle but important aspect of ML, referring to the unintended use or influence of data (that should not be available at the time of prediction) during the training process. This often occurs when the features used for training hide within themselves the result of the prediction, resulting in an overestimation of performance of the model during validation (Chiavegatto Filho et al., 2021). Due to the subtleties with which this can occur, avoiding data leakage is challenging and should be evaluated on a case by case basis. Important aspects for consideration here have been discussed previously (Wirbel et al., 2021) and include (1) data filtering that is influenced by the target label and (2) the splitting of dependent data (e.g., replicates and time-series data points) across training and validation sets. The use of an externally generated test data set (handled separately from the training set) for additional validation checks can help (Oyetunde et al., 2019; Wirbel et al., 2021), though data leakage is seldom discussed in microbial omics papers that use SML. We urge future authors in this space to consider including at least a statement on leakage assessment in studies based on SML.

### Applications of Molecular Microbial Ecology–Machine Learning for Environmental Challenges

#### Microbes as Environmental Biosensors

Anthropogenic impacts are motivating the development of cost-effective and scalable environmental bioassessment methodologies (Fruehe et al., 2021). Microbes have long been recognized as potential *in situ* biosensors for following human impacts (Su et al., 2011), allowing for highly accurate quantitative SML predictions of the perturbation. Indeed, metataxonomic data can be valuable for the prediction of a variety of environmental contaminants (Table 1), spanning from relatively inert plastics (Li et al., 2021) to petroleum hydrocarbons [which illicit strong responses with detectable influences even after the pollutant is degraded and undetectable by conventional measures (Smith et al., 2015)]. Hydrocarbonoclastic indicator species have also been identified as key biosensors in ML-based bioprospecting of hydrocarbon seepage from subsurface reservoirs and can improve the likelihood of success in drilling for new assets (de Dios Miranda et al., 2019; Chitu et al., 2022). The same approach is also being explored as the potential early-warning indicators of leakage from hydrocarbon transport lines (Shaheen et al., 2011). Indeed, the SML of microbial fingerprints has even demonstrated reasonable predictions (accuracies of 72–85%) of the future production of hydrocarbon reservoirs (using metataxonomic input) (Zijp et al., 2021) which can facilitate decision-making for enhanced asset management. These approaches thereby have real potential for reducing the carbon footprint and ecological impact of upstream oil and gas activities.

#### Microbes as Predictors of Environmental Status

Microbes have proved valuable as ecological assessment indicators in multiple diverse environments (Astudillo-García et al., 2019; Glasl et al., 2019; Hermans et al., 2020; Chen et al., 2021). Moreover, improvements in sequencing technologies are facilitating the upscaling and deployment of omics-based ML for more ambitious environmental monitoring and mitigation applications (Wang et al., 2021). These indicators can reveal important relationships for land management, when conventional field measurements are unhelpful (Chang et al., 2017). Indeed, the SML of microbial 16S rRNA abundances can directly predict soil productivity in arable land and risks posed for agriculture (Yuan et al., 2020). USML is routinely applied *via* ordination techniques to establish the organization of microbiome data in relation to their environmental parameters. However, in instances where conventional ordinations fail to determine clear relationships, SML may still yield community subpopulations that can serve as predictors for environmental parameters and processes of interest. For example, the influence between temperature and key phosphate and glycogen-accumulating organisms involved in the enhanced biological phosphorous removal processes of a set of wastewater treatment plants (WWTPs) in South Korea was identified using an SML approach, resulting in findings with clear implications for WWTP design and operation (Oh and Kim, 2021). Additionally, the SML of metabarcoded environmental DNA (eDNA) can provide superior performance for environmental quality monitoring over conventional bioindicator values for marine aquaculture monitoring (Fruehe et al., 2021). Furthermore, RF learning of eDNA has been shown to outperform conventional taxonomy-based biotic indices assessments (Cordier et al., 2018). Biodiversity in microbial communities can also be a useful proxy to assess the environmental impact of anthropogenic perturbations through changes in biotic indices (Aylagas et al., 2017). In these ways, SML is a useful means to improve environmental monitoring programs.

#### Predicting Sample Origin With Microbiological Data

The predictive power of ML for monitoring environmental status also enables sample origin to be established (Raza et al., 2021). Microbial metrics have proved to be exceptionally sensitive indicators of human impacts on freshwater environments (Liao et al., 2018). Indeed, *via* ML modeling, the partitioning of microbes along complex anthropogenic xenobiotic gradients from urban and agricultural runoffs is sufficient to identify the origin of water samples from the 30 most abundant taxa (Wang et al., 2021) and is able to resolve sample origin depth and local salinity in the Baltic Sea (Alneberg et al., 2020). Such origin tracing carries the potential to inform for public health by accurately predicting the origins of fecal contaminants in public waters (Chen et al., 2021; Raza et al., 2021) and the source of food-borne pathogen outbreaks (Wheeler, 2019). The ability to identify sample origin sources is likely to be of critical importance moving forward for tracing runoffs from agricultural and industrial entities to ensure compliance with environmentally mindful legislation. It will be interesting to see whether this sort of tracing application will lend itself to following waterbodies in other settings, or indeed, other mobile elements within the environment (forensic analysis of migratory animals under conservation management, for example). Given the perceived stability in the gut microbiome, it is possible that this approach could also be extended as a biological tagging approach for following animal populations at the center of conservation efforts.

#### Supporting Environmental Meta-Analyses and Data Mining

The high volumes of omics data are enabling large-scale meta-analyses (Zeller et al., 2014) that can provide a global view of microbial roles within major environments (Ramirez et al., 2018; Wu et al., 2019; Yuan et al., 2020). However, several challenges arise in such studies owing to non-standardized sample collection, extraction methods, and primer choice (Ramirez et al., 2018). Additionally, technicalities of sequencing platforms, variable library sizes, and environmental confounders can reduce concordance across omics studies (though SML is alleviating this issue) (York, 2021). ML tools are well suited for uncovering patterns within these challenging data collections. For example, a meta-analysis of soil microbiomes with SML was able to reveal microbiological indicators for predicting propensity for *Fusarium* wilt (Yuan et al., 2020), an agriculturally important pest. Additionally, a meta-analysis of global soil (Ramirez et al., 2018) and WWTP (Wu et al., 2019) communities provided macroecological insights into the microbial biogeography communities and confirmed the importance of the rare microbiome members as bioindicators. There remains significant scope for standardizing the workflows in both omics and SML. Such standardizations are crucial to mitigating common pitfalls; these enhance reproducibility and promote meta-analyses and data mining. An important limiting factor here is that many data sets are unavailable, uploaded to repositories without raw data or lacking metadata descriptions. This issue has been raised before (Ramirez et al., 2018) and impedes otherwise valuable work. For instance, bioprospecting of biosynthetic gene clusters with SML-based omics data mining can yield proteins with biotechnological potential (Correia and Weimann, 2021) for bioremediation, biodegradable plastic production, and sustainable biofuels (Haque et al., 2020; Keasling et al., 2021). We therefore urge that omics data sets be uploaded in their raw form with metadata made available.

#### Supervised Machine Learning of Microbial Omics Data to Address Climate Change

The collective effects of anthropogenic perturbations are driving the consequences of climate change (notably, losses of ecosystem function, services, biodiversity, and habitat) at unprecedented rates (Giuliani et al., 2017). The actions of microbial communities are implicitly tied to geochemical cycling, global water chemistries, nutrient availabilities, and soil/plant health (Gorbushina and Krumbein, 2000; Falkowski et al., 2008; Lian et al., 2008; Dong, 2010; Panke-Buisse et al., 2014). Microbes are thereby drivers of numerous ecosystem services on which the global population relies (Marco and Abram, 2019). Understanding microbe–ecosystem interactions and functions is therefore central to their utilization in ecological models and biotechnologies for intervening on climate change. The generation of high-resolution spatiotemporal dynamics data and incorporation of different omics data sets can provide important insights into the molecular mechanisms behind climate changes responses and improve the accuracy of forecasting models (Herold et al., 2020; Layton and Bradbury, 2021). Together with their ubiquitous nature, the core roles of microbial communities afford us with a broad framework for potential microbiological tools with which the fundamental impacts of global climate change can be understood, monitored, predicted, and conceivably, mitigated. The short generation times of microbial community members and their predictable changes following changing environmental parameters (Larsen et al., 2012) open the possibility for their use as early-warning indicators of climate change-led impacts on macroecological networks (Shah et al., 2022) before further biodiversity loss is observable on the macroscale. Conversely, microbial contributions to climate change *via* carbon cycle-climate feedback and N_2_O production (Bardgett et al., 2008) are an ideal candidate for predictive SML modeling and intervention. Indeed, predictive models from microbial omics data have also shown utility across a range of climate change-linked phenomena, including browning (Fontaine et al., 2021), eutrophication (Glasl et al., 2019), harmful algal blooms (Hennon and Dyhrman, 2020), and arability of soils (Chang et al., 2017; Hennon and Dyhrman, 2020; Yuan et al., 2020). omics in soil-plant, subsurface, and aquatic microbiomes is also central to making inroads in the development of carbon capture and sequestration (CCS) biotechnologies (Schweitzer et al., 2021). It will be interesting to see whether such developments benefit from SML-based modeling, which could prove useful for establishing taxa and metabolisms that predict stability and sequestration rates in CCS systems. Therefore, SML modeling can facilitate the establishment and optimization of carbon fluxes in microbial communities (particularly for the poorly characterized deep subsurface microbiome) and may also help to bridge bioenergy production to CCS, which is considered essential for many climate change mitigation plans (Hanssen et al., 2020). At present, the ability of microbes to inform on, and forecast, climate change impacts *via* ecological monitoring programs is perhaps the most immediately applicable area for the SML of microbial omics in climate change research. In this way, microbes can assist decision-makers for sustainable policies and intervention measures to ensure food security and maintain ecosystem services before further ecological detriment occurs (Cordier et al., 2021; Shah et al., 2022). The potential future applications in this space, however, are vast and may be key for realizing goals in global-scale climate management and engineering against climate change.

## Concluding Remarks and Future Perspectives

Machine learning is a powerful toolbox for drawing meaningful biological insights from large multidimensional microbial data. Here, we discussed how SML can contribute to environmental challenges by valorizing microbial community data sets. The predictive potential of interfacing omics and SML has opened exciting new avenues for managing environmental pollution and status. The ability to identify key species and functional elements can be expected to accelerate biotechnological developments with implications for environmental intervention (such as bioremediation). Through the interface of these important disciplines, we are rapidly advancing our view of global microbiome and the ecological impacts from human activities.

This nascent, but fast-evolving, application area for ML has several notable opportunities which are yet to be exploited. Metataxonomics-centric ML efforts have dominated this space, but has yet to apply long-read and metagenome-assembled genomic data for feature set development in this research area. Additionally, several advanced systems-level techniques (metaproteomics, metabolomics, and in particular, integrative omics) remain at much earlier stages of development compared with DNA sequencing-based approaches and are consequently lagging in this arena. ML tools will likely become integral to pipelines for these advanced omics methodologies. We foresee SML becoming a routine complement to conventional statistics and expect that this will key for revealing the often-overlooked rare microbiome. As omics approaches continue to advance, and sample costs reduce, we can expect to see a rise in the application of promising DL architectures at this interdisciplinary interface. DL tools will no doubt prove indispensable in data mining the ever-increasing public omics repositories and represent an exciting means to address feature engineering challenges *via* unsupervised feature extractions.

## Author Contributions

JM: structure of manuscript, figure design and production, literature review, manuscript writing, population of table, and revisions. MC: initial draft of manuscript, figure design, and literature review. AM: literature review, population of table, figure design, and development of content. AH: structure of the manuscript, secured funding, manuscript review, and development of content. JD: conceptualize the manuscript, manuscript review, and development of content. All authors contributed to the article and approved the submitted version.

## Conflict of Interest

The authors declare that the research was conducted in the absence of any commercial or financial relationships that could be construed as a potential conflict of interest.

## Publisher’s Note

All claims expressed in this article are solely those of the authors and do not necessarily represent those of their affiliated organizations, or those of the publisher, the editors and the reviewers. Any product that may be evaluated in this article, or claim that may be made by its manufacturer, is not guaranteed or endorsed by the publisher.
